# Deep-time paleogenomics and the limits of DNA survival

**DOI:** 10.1126/science.adh7943

**Published:** 2023-10-05

**Authors:** Love Dalén, Peter D. Heintzman, Joshua D. Kapp, Beth Shapiro

**Affiliations:** 1 Centre for Palaeogenetics, Svante Arrhenius väg 20C, SE-10691 Stockholm, Sweden.; 2 Department of Zoology, Stockholm University, SE-10691, Stockholm, Sweden.; 3 Department of Bioinformatics and Genetics, Swedish Museum of Natural History, SE-10405 Stockholm, Sweden.; 4 Department of Geological Sciences, Stockholm University, SE-10691, Stockholm, Sweden.; 5 Department of Biomolecular Engineering, University of California Santa Cruz; Santa Cruz, California, 95064, USA.; 6 Department of Ecology and Evolutionary Biology, University of California Santa Cruz; Santa Cruz, California, 95064, USA.; 7 Howard Hughes Medical Institute, University of California Santa Cruz; Santa Cruz, California, 95064, USA.

## Abstract

While most ancient DNA studies have focused on the last 50,000 years, paleogenomic approaches can now reach into the early Pleistocene, an epoch of repeated environmental changes that shaped present-day biodiversity. Emerging deep-time genomic transects, including from DNA preserved in sediments, will enable inference of adaptive evolution, discovery of unrecognized species, and exploration of how glaciations, volcanism, and paleomagnetic reversals shaped demography and community composition. In this review, we explore the state-of-the-art in paleogenomics and discuss key bottlenecks, including technical limitations, evolutionary divergence and associated biases, and the need for more precise dating of remains and sediments. We conclude that with improvements in laboratory and computational methods the emerging field of deep-time paleogenomics will expand the range of questions addressable using ancient DNA.

## Teaser

A review of research and discovery opportunities by leveraging paleogenomes from the Early and Middle Pleistocene.

The Pleistocene epoch (approximately 2.6 million years ago; Ma, to 10 thousand years ago; ka) was a time of considerable environmental upheaval that shaped the present worldwide distribution of biodiversity. Environmental changes during the Pleistocene included cyclical fluctuations in global temperatures and precipitation patterns, advances and recessions of high-latitude ice sheets, and dramatic changes in sea-level, together with large-scale volcanism, paleomagnetic reversals, and the global spread of humans (1). These events altered habitats around the world, driving changes in resource availability and ecological community composition.

The rich fossil record of the Pleistocene has been instrumental for testing hypotheses about correlation between these environmental changes and biodiversity dynamics, especially at high latitudes where the cold climate favors fossil preservation. This is particularly true for the Late Pleistocene (126–11.7 ka), thanks to fine-scale inferences enabled by ancient DNA preserved in fossils dating to this period. Such inferences have allowed insights into population turnover (2–4) and inter-species gene flow (5)–processes that are invisible to traditional paleontological techniques–and shown that demographic trends in large mammals closely track available habitat (6).

**Fig. 1.**
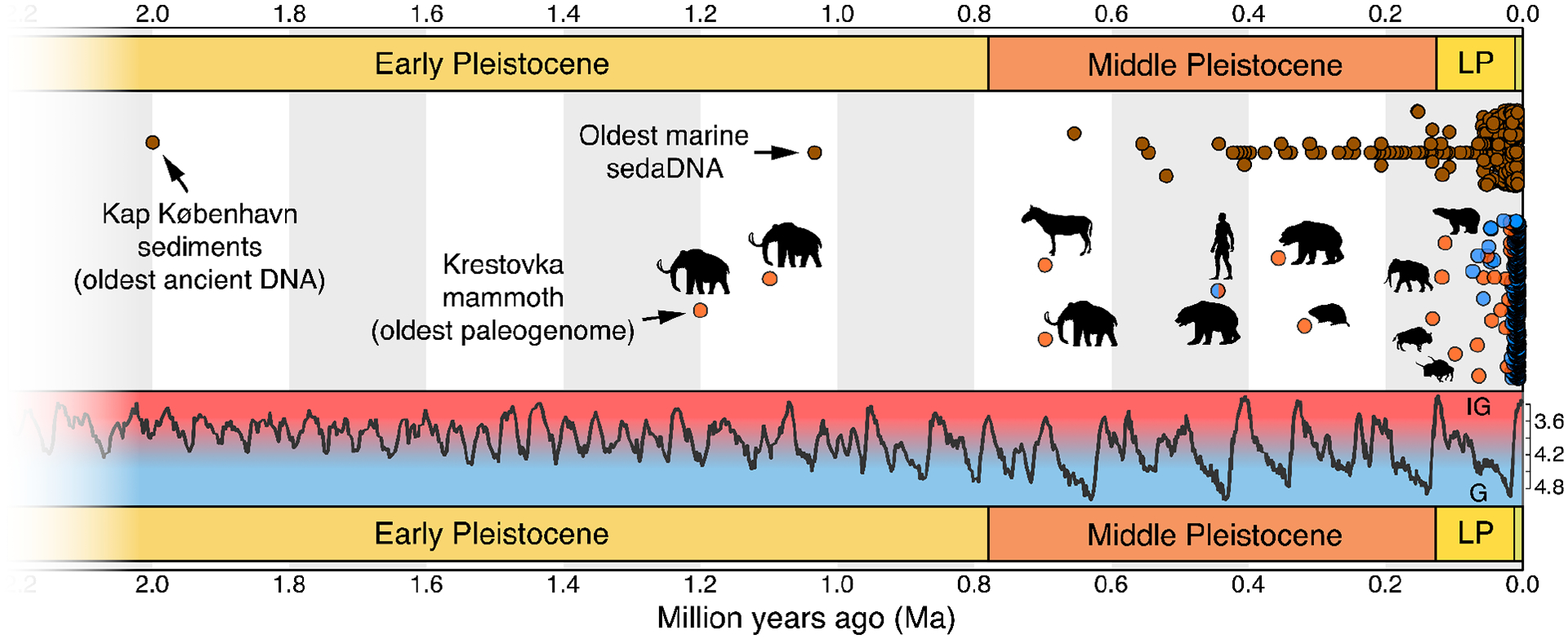
The temporal distribution of ancient DNA studies to date highlights gaps and opportunities for deep-time paleogenomics and sedimentary ancient DNA. Circles in orange are non-human animal paleogenomes, in blue are hominin paleogenomes, and in brown are sedimentary ancient DNA records. Most ancient DNA studies fall within the last 50 ka and the most recent glacial cycle. The climate curve is based on benthic **δ**^18^-Oxygen measurements (per mil, ‰; LR04 stack from (42). Sedimentary ancient DNA data are from the AncientMetagenomeDir (v23.06.0, 58) and von Eggers et al. (v1, https://doi.org/10.5281/zenodo.6847522), with metabarcoding records older than one million years excluded. Paleogenomes older than 100 ka are annotated with a silhouette of the study taxon, with the deep-time paleogenomes including a 130 ka steppe bison (36); 330 ka collared lemming (40); 360 ka cave bear (9); 430 ka cave bear and hominin (35, 59); 700 ka horse (8); and 700 ka, 1.1 Ma, and 1.2 Ma mammoths (10). Silhouettes are from PhyloPic (https://beta.phylopic.org/) and are in the public domain with credits to Zimices (mammoth, two bison) and Robert Bruce Horsfall (horse). LP: Late Pleistocene; IG: Interglacial; G: Glacial.

Technical advances in DNA recovery have extended the ability to make these inferences deeper into the Pleistocene. DNA from bones and teeth that are several hundreds of thousands of years old (7–9) and beyond one million years old (10) has now been recovered and analyzed (Fig. 1). Such deep-time paleogenomes, which we consider here to refer to genomes assembled from organisms that lived during or earlier than the Middle Pleistocene, i.e. >126 ka, are still rare because post-mortem processes lead to successive degradation of DNA molecules into increasingly small fragments, making DNA recovery more difficult with age. Early and Middle Pleistocene DNA has, however, been recovered from remains and sediments in high-latitude permafrost (10–14) and lower latitude caves (15, 16), suggesting that deep-time genomics is feasible in ideal preservation environments. Here, we explore the current state-of-the-art in deep-time paleogenomics research, the key obstacles preventing wider adoption, and scientific questions that deep-time paleogenomics can address.

## DNA persistence into deep time

DNA does not survive indefinitely, but it does survive for significantly longer than the earliest models predicted. In 1993, Lindahl estimated that hydrolytic depurination would lead to complete degradation of DNA molecules within several tens of thousands of years (17). This limit has since been exceeded, and DNA is regularly recovered from remains and sediments that date to within the last 100 ka. As of May 2023, the oldest reconstructed paleogenome is from a permafrost-preserved mammoth dating to 1–2 Ma (10) and the oldest isolated DNA is from ~2 Ma sediment from northern Greenland (11), but the maximum age of recoverable and useful DNA molecules–those that are long enough to retain information–remains uncertain.

**Fig. 2.**
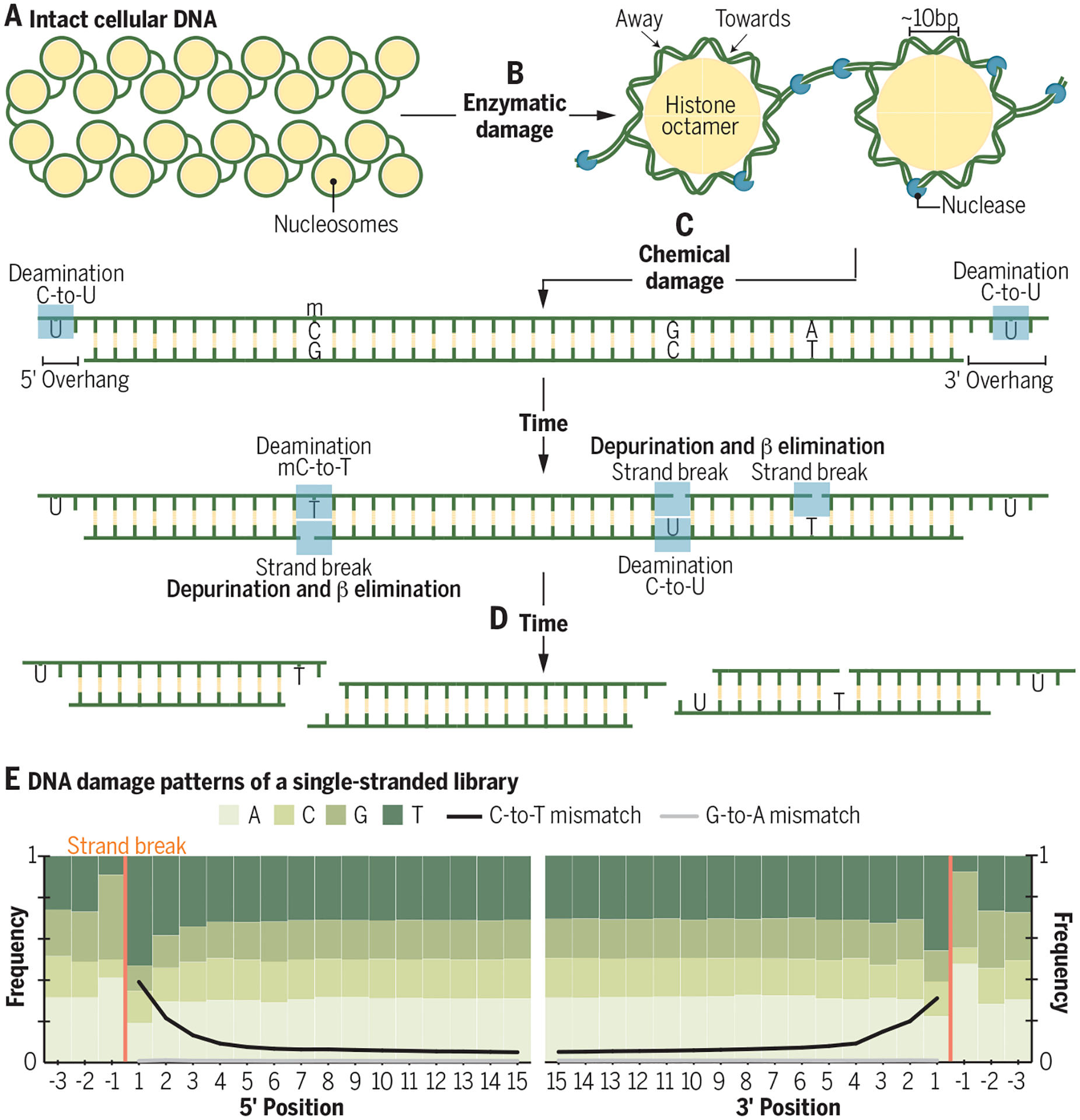
DNA fragmentation and degradation begins after death and continues until fragments are too short to be useful. (**A**) The integrity of megabase length DNA is maintained by a cell’s enzymatic repair machinery and, in eukaryotic genomes, packaged in histone-DNA complexes. (**B**) Following death, repair stops and DNA damage begins to accumulate. Nucleases and microorganisms cleave DNA in labile regions between nucleosomes and when the DNA backbone faces away from histones. (**C**) Over time, chemical damage also accumulates. Cytosine bases are converted to uracil and methylated cytosines are converted to thymines (by deamination). Cytosines are particularly vulnerable to deamination in single-stranded regions such as in overhanging regions at DNA termini, but deamination is possible in some double-stranded contexts. Fragmentation occurs after the loss of purine bases (depurination), creating abasic sites that can be cleaved by β elimination. Depurination and β elimination create a region of single-stranded DNA, which leaves cytosines vulnerable to deamination. . (**D**) Given enough time, DNA molecules will become too short to be identifiable. (**E**) A summary of base and mismatch frequencies along the initial 15 5’ and 3’ bases of reads generated using a single-stranded DNA library protocol. Depurination leads to overrepresentation of adenine and guanine bases adjacent to strand breaks. C-to-T mismatches are elevated near read ends and observed throughout damaged reads. While 3’ G-to-A mismatches are observed in double-stranded libraries, single-stranded libraries show a C-to-T signal at both ends by retaining the native termini of the molecules.

DNA begins to degrade immediately following organismal death, initially through microbial and endogenous nuclease activity (Fig. 2). In nuclear DNA, strands are cleaved in labile regions of histone-DNA complexes, resulting in a ~10-base periodicity in the distribution of the lengths of recovered molecules (18). The primary chemical mechanism of DNA fragmentation is hydrolytic depurination. This process removes adenine or guanine bases, creating abasic sites that can be cleaved by β elimination (19; Fig. 2C), and leading to purine overrepresentation adjacent to strand breaks (20; Fig. 2E) and interior gaps (21). Hydrolytic deamination, another common form of chemical damage, converts cytosine to uracil and is observed as thymine in sequencing data, or “C-to-T transitions” (Fig. 2C). Deamination occurs primarily near strand ends and in single-stranded DNA (17, 21, 22; Fig. 2E). DNA crosslinking (19, 22) and oxidative damage (20, 23) also occur but are observed less frequently than depurination and deamination. These typical damage patterns can be used to bioinformatically corroborate the authenticity of recovered ancient sequences and, to reduce their impact on sequence accuracy, can be identified and removed from ancient DNA data sets using standard bioinformatic approaches.

Recovery of increasingly old and damaged DNA is possible in part due to technical advances in the laboratory. Ancient DNA isolation methods are optimized to recover both short DNA molecules and molecules containing nicks and gaps. Extracted molecules are prepared for sequencing by ligating platform-specific adapters to either double-stranded or single-stranded DNA. Single-stranded approaches to genomic library preparation (24, 25) convert natively single-stranded DNA as well as double stranded DNA and more effectively convert molecules containing nicks and gaps compared to double-stranded approaches. DNA extracts are also often treated with uracil DNA glycosylase and endonuclease VIII to reduce deamination damage by removing uracil bases (26). While this approach reduces damage-induced errors in the resulting sequencing data, it also cuts the DNA backbone at abasic sites and shortens the recovered molecules by 5–10 nucleotides (26). As deep-time DNA molecules are short, often <35 bases (15), this may reduce the proportion of useful endogenous DNA.

The short nature of deep-time DNA molecules makes them prone to spurious alignment and reference bias (27), complicating genome assembly and analysis. For example, ancient DNA data sets comprise both endogenous DNA from the target organism(s) and introduced exogenous DNA. These categories of molecules can be separated by identifying each read via taxonomic assignment, which can be problematic if the ancient organism has no close living relative to act as a genomic reference. Lack of a close reference, reference bias, and errors introduced by damage will also impede variant and consensus calling. Bioinformatic approaches mitigate these challenges by directly modeling DNA damage and/or bias as part of genotyping (28), or considering only substitutions that are not impacted by cytosine deamination. Reference genomes can also be modified to create artificially closer references, such as a “Neandertalized” version of the human reference genome for reference-guided mapping of Neandertal reads (29). Genotype likelihoods rather than strictly called genotypes can also be used during downstream analysis, although imputation-based analytical methods may be inappropriate for deep-time data sets if ancient genomic diversity is not represented in existing reference panels.

## Research opportunities arising from deep-time DNA

### Speciation and evolution

**Fig. 3.**
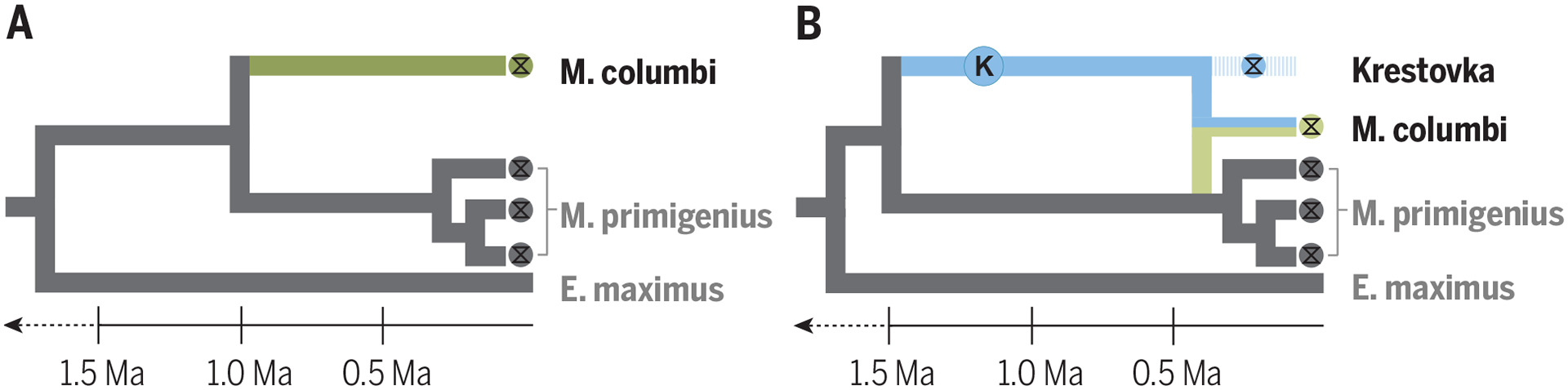
Deep-time paleogenomes provided new understanding of the evolutionary history of mammoths. Paleontological hypotheses assumed that the *M. columbi* lineage evolved after early divergence from *M. primigenius* (**A**), however, isolation of a deep-time paleogenome from the Krestova mammoth (blue circle) revealed that *M. columbi* emerged more recently and following admixture with the Krestova mammoth lineage (**B**).

Speciation is not always a simple process of cladogenesis followed by reproductive isolation. Instead, modern and paleogenomic data have shown that interspecific hybridization is surprisingly common and perhaps driven in part by repeated habitat redistribution associated with glacial cycles (5, 9, 10). For example, brown bears and polar bears hybridize today and also hybridized during previous glacial and interglacial periods (30, 31). Recently, polar bear and cave bear paleogenomes dating to up to 360 ka revealed that all living brown bears derive a portion of their ancestry from admixture with these other bear lineages–evolutionary events that were invisible without these paleogenome (9, 32). Similarly, a mammoth paleogenome dating to the Early Pleistocene revealed that Columbian mammoths originated after hybridization between two distinct ancient mammoth lineages (10; Fig. 3). Taxonomically diverse deep-time paleogenomes could clarify the timing, rate, and extent of genomic introgression episodes and their role in evolution. Paleogenomic data from species that went extinct during the Early and Middle Pleistocene, such as short-faced hyenas, European jaguars, and the enigmatic *Xenocyon* canids, could shed light on whether these taxa contributed to the genetic make-up of living carnivores. Deep-time paleogenomes could also identify unknown “ghost” lineages that contributed to species’ ancestries, as exemplified in the paleogenomic characterization of the Krestovka mammoth (10; Box 1, Fig. 3).

Deep-time DNA can also reveal genomic snapshots of a species’ entire evolutionary story (Box 1). As many temperate and cold-adapted birds and mammals trace their origin to the Early and Middle Pleistocene (33, 34), paleogenomes from these species could correlate evolutionary changes to specific environmental perturbations, such as transitions between climate regimes or community reshuffling. The process of speciation can be investigated as it happens, exploring founder event bottlenecks and testing whether speciation occurred through strict allopatry or gradually with post-divergence gene flow. As deep-time paleogenomes tend to occupy basal phylogenetic positions within their clades, they can also provide important calibrations for estimating rates of molecular evolution. For example, paleogenomic data from a Middle Pleistocene hominin from Sima de los Huesos in present-day Spain confirmed hypotheses from Late Pleistocene genomes that Neandertals and Denisovans diverged during the early Middle Pleistocene (35), whereas the inclusion of a ~700 ka horse paleogenome in the equid phylogeny pushed the estimated time for the origin of living equids to more than twice as old as previously hypothesized (8).

Deep-time paleogenomes can also be used to test hypotheses about relationships between species and how derived forms are related to earlier forms. An outstanding question in paleontology is whether fossil morphospecies are true species, synchronous ecomorphs, or chronospecies that were direct ancestors of succeeding species. A paleogenomic study of ancient North American bison dating to ~130–110 ka, for example, showed that two samples exhibiting extreme size dimorphism and representing supposedly distinct species–the longhorn bison and the steppe bison–actually belong to the same lineage that dispersed into North America only a few tens of thousands of years earlier (36). Conversely, deep-time paleogenomics can also give context to species for which we have only limited remains, such as Denisovans (35).

Finally, paleogenomes across deep time-scales will also make it possible to explore aspects of adaptive evolution. At the most basic level, deep-time genomes can help identify when adaptive mutations arose. For example, comparative analysis of mammoth paleogenomes ranging from a few thousand to more than a million years old identified genes associated with hair and skin development, fat storage and metabolism, immune system function, and body size that evolved in that lineage within the last 700 ka (37). Paleogenomes will also allow exploration of how the rate of protein-coding changes varies over time, such as in conjunction with past changes in climate, as well as to assess when genomic deletions arose and the rate of positive and purifying selection in introgressed genomic regions.

### The impact of glacial cycles on biodiversity

Nearly all ancient DNA studies to date have for practical reasons focused on Late Pleistocene or more recent materials (Fig. 1). Thus, our current understanding of evolutionary processes during the Pleistocene mostly relies on more traditional approaches, including morphometrics, stable isotope analysis, and pollen records. This is despite the fact that the majority of the Pleistocene glacial oscillations occurred during the Early (2.6 Ma - 780 ka) and Middle (780 – 126 ka) Pleistocene sub-epochs, which are now accessible with deep-time paleogenomes.

A special attribute of the Pleistocene is the change in periodicity of glaciations from ~40 ka cycles to ~100 ka cycles that occurred 1.2–0.7 Ma (38) (Fig. 1). This change isolated temperate species in glacial refugia for longer periods, providing more time for local adaptation and increasing the rate of population divergence. Biological communities may also have been reshuffled following this periodicity change, as the longer and higher amplitude glaciations allowed sufficient ice sheet accumulation for the Bering Land Bridge to form, making land dispersal between Eurasia and North America possible.

Since the change in glacial periodicity, the dominant pattern has been cycles of long glaciations separated by short warm interglacials. This pattern is believed to have driven the demography and range dynamics of many species (39). Long interglacials, for example, have been correlated with bottlenecks in cold-adapted taxa (40) and expansion and speciation in warm-adapted taxa (41). Of particular interest is the unusually long interglacial that occurred 420–370 ka (Marine Isotope Stage 11) (42). Paleogenomes from individuals that lived during and earlier than this long bottleneck could test these hypotheses and reveal evolutionary changes that may have been overwritten by subsequent genetic bottlenecks.

### Inference of ancient ecosystems

Above, we describe insights potentially derived from DNA extracted from remains of individuals that lived during the Middle Pleistocene and earlier. However, the advances that enable deep-time paleogenomics also make it possible to reconstruct entire deep-time ecological communities. To date, only five studies have attempted to use sedimentary ancient DNA to reconstruct plant and/or animal communities dating to the Middle Pleistocene or older: Kjær et al (11) reconstructed components an Early Pleistocene interglacial ecosystem from a sediment core extracted from the present day polar desert in northern Greenland, Armbrecht et al (43) reconstructed an Early to Middle Pleistocene marine ecosystem from Iceberg Alley in the Southern Ocean, Courtin et al (12) reconstructed a Middle Pleistocene interglacial ecosystem from a permafrost megaslump in Eastern Siberia, and Willerslev et al reconstructed Middle Pleistocene plant communities from sediments collected below the Greenland ice sheet (14) and from coastal Siberian permafrost (13). Among these, Kjær et al and Armbrecht et al enriched libraries for sequences of interest via hybridization to synthesized baits designed to target Arctic or Antarctic taxa. In contrast to metabarcoding methods, which use targeted PCR amplification, hybridization-based targeted enrichment can capture molecules of any length and are therefore powerful even when preserved molecules are short. While this approach is limited today to capturing sequences that are genetically similar to other known taxa, methodological improvements in hybridization capture is a ripe area of research that will no doubt expand access to deep-time sedimentary DNA.

Deep-time sedimentary DNA research will allow better understanding of the effect of glacial-interglacial transitions on community composition. Reconstructions of communities spanning the transition into the present Holocene, for example, have revealed rapid biological turnover that closely tracked abiotic changes (44, 45). Comparison with older transitions will test whether patterns are predictable or idiosyncratic, whether some species or communities are more resilient to environmental upheaval than others, and whether some transitions or events leave lasting signatures on community biodiversity.

Reconstructions of communities that thrived in past warm interglacials may provide insight into the potential composition of communities in a future, warmer world (11), and improve our understanding of how ecosystem-level interactions among species evolve and are maintained. They also enrich our understanding of these extinct ecosystems beyond what is knowable from the fossil record. Deep-time sedimentary DNA from northern Greenland, for example, revealed a mastodon or mastodon-like animal was part of the Early Pleistocene community (11) despite that no fossil remains from such an animal have been discovered. Deep-time sedimentary DNA can also reveal past connectivity among populations, as in a recent study of Late Pleistocene sedimentary DNA from a cave in Mexico that linked an extinct population of black bears to living populations in eastern North America (46). As technologies improve, in particular those that allow increasingly sensitive targeted enrichment, we envisage deep-time sedimentary DNA as a powerful tool to explore the ecological and evolutionary consequences of environmental change on community-level biodiversity.

## Future research to enable deep-time DNA

It has been shown that DNA can survive in ideal preservation conditions into at least the Early Pleistocene. The next phase of deep-time DNA research is to expand the taxonomic, geographic, and temporal range of recovered and authenticated deep-time DNA. This challenge presents new research opportunities in the field, at the bench, and bioinformatically.

Deep-time genomics is today mostly conducted on substrates with optimal DNA preservation such as those derived from permafrost or caves. However, more efficient approaches to recover ancient DNA molecules will continue to expand the range of samples and substrates suitable for analysis. Today, methods for DNA extraction and library conversion do not recover all potentially preserved DNA molecules. For example, Kjaer et al (11) found that DNA adsorbed preferentially to clay mineral surfaces compared to non-clay surfaces, and in particular to the clay mineral smectite, which can bind 200 times more DNA than quartz and is a common mineral in terrestrial samples. Their best performing extraction protocol recovered 40% of DNA bound to quartz and only 5% of DNA bound to smectite, suggesting the majority of DNA was inaccessible. While anecdotal, this observation points to several opportunities for improving deep-time DNA research, including using mineralogical characterization to identify the most promising sites for deep-time sedimentary DNA recovery and refining experimental approaches to recover DNA bound to all mineral surfaces. In the absence of improved methods to release bound DNA, microscopic evaluation of sedimentary samples will improve the efficiency of DNA recovery. Massilani et al (47), for example, showed DNA preserved in cave sediment is concentrated in micro-scale particles, especially fragments of bone and feces preserved within the substrate.

Library conversion protocols could also be made more efficient. Optimized library conversion protocols use enzymatic ligation and polymerization, but ancient DNA extracts contain inhibitors as well as molecules with uncharacterized DNA damage. Although we can convert as little as 100 picograms of DNA into libraries using the Santa Cruz method (25), library preparation has been shown to typically convert only around 10–50% of extracted DNA (21), suggesting that most recovered molecules are lost at this experimental step. Improvements in library preparation may include engineering more robust enzymes to combat inhibitors or developing protocols that incorporate enzymatic repair during library conversion. Additionally, reducing reliance on ligase and polymerase steps through alternative enzymatic strategies, bioorthogonal chemistry, or native DNA sequencing may offer new approaches to convert currently unsequenceable DNA molecules.

Many species that are obvious targets for deep-time DNA research are extinct, and some, such as *Xenocyon* canids and basal members of the elephant and horse families, have no evolutionarily close living relative for which an ideal reference genome can be produced. This presents challenges to ancient DNA authentication and identification as well as to reference-guided genome assembly. Although the average fragment length of deep-time DNA sequences is short, it may be possible to generate de novo assemblies from ancient extracts by capitalizing on methods that use chromosome conformation capture to retain proximity information useful to link short reads within a chromosome (48). Approaches that sequence DNA in situ (49) are also promising, but in early stages of development. Improvements in bioinformatic processing will also benefit eukaryotic paleogenomic reconstruction and variant calling. Recently, microbial genomes were assembled from DNA recovered from relatively recent paleofecal samples (50) and from archaeological dental calculus dating to as old as 100 ka (51), suggesting a bioinformatic path toward de novo assembly of some small paleogenomes. While this approach is not likely to apply to complex eukaryotic genomes, other bioinformatic approaches can improve the accuracy of these assemblies from short read data. Replacing linear single-species reference genomes with multi-species variation graphs that incorporate variants from several genomes (52), for example, can increase the number of reads that map to a reference genome. This approach has the additional benefit of allowing variation among indel lengths as well as among nucleotides. Iterative assembly approaches, such as the mapping-iterative-assembler used to generate the first Neanderthal mitochondrial genome (53) may improve mapping to more complex genomes. Finally, as reference-based taxonomic assignment is always limited to sequences deposited in public databases, the ongoing population of these databases will continue to improve robust identification of DNA recovered from Early and Middle Pleistocene remains and sediments.

A considerable challenge for studies of deep-time DNA is to know how old samples are so that they can be placed into broader evolutionary and geological contexts. As most ancient DNA to date is from organisms that lived relatively recently, it is usually possible to estimate their age directly using radiocarbon dating. However, the short radioactive half-life of carbon-14 means that age estimates are often unreliable if organisms lived more than ~50 ka. For samples older than ~50 ka, alternative methods are necessary. Trapped charge dating methods, such as electron spin resonance (ESR) for tooth enamel or luminescence approaches for minerals such as quartz and feldspar, can provide age estimates for samples dating throughout the Pleistocene, but require that sediments have remained undisturbed since burial (for a review see 54). When proteins are preserved, the extent of amino acid racemization, hydrolysis, and decay can also estimate time since death, although amino acid “clocks” vary among species and localities (54).

In some cases, paleoenvironmental, geological, and geophysical markers can provide clues about a sample’s age. A fossil might be found in the Arctic with other paleoecological proxies that suggest a warm and wet environment, for example, indicating that the animal lived during a previous interglacial, or in sediments with reversed polarity, suggesting that it lived prior to the last paleomagnetic reversal some 780 ka. In some environments, tephra beds–layers of fine, settled, volcanic ash–can be dated by methods including glass fission-track and argon-argon dating. Tephra beds, which can be detected even when present in only microscopic amounts (55), have been particularly important in dating sediment cores, but can also provide contextual clues about the age of samples found *in situ* at sites where the tephra is present. As volcanic eruptions were common throughout the Pleistocene, improved tephrochronology for the Early and Middle Pleistocene will help place deep-time DNA into a chronological context.

Other approaches to dating deep-time genomes might rely on the predictable nature of evolutionary change in organisms. Molecular clock methods infer the age of paleogenomes by estimating the amount of “missing” evolution along a phylogenetic branch leading to the paleogenome, often called “branch shortening” (56). Because the accumulation of mutations is approximately constant over time, the differences between these branch lengths should correspond to the number of generations that separate the represented paleogenome from extant or more recent individuals. To translate missing generations into calendar time, however, the branch shortening approach requires either an independent fossil calibration or an estimate of generation length. For many lineages that lived during the Early and Middle Pleistocene, dated ancestral fossils are few and, with no close living relatives, estimates of generation time would be imprecise. Variation among evolutionary rates between distantly related lineages may also reduce the power of a comparative molecular dating approach. Nonetheless, development of approaches that use genomic information to estimate the age of paleogenomes and their evolutionary relationships to other species is a rich area for future research.

## Conclusion

The next decade will bring continued technical advances that will increase the taxonomic and geographic range of deep-time paleogenomes and deep-time ancient sedimentary DNA data sets. Most crucially, new insights into what substrates are likely to preserve deep-time ancient DNA as well as refined approaches to release DNA bound to biological or mineralogical matrices will increase the number and taxonomic range of recoverable deep-time paleogenomes. These will need to be placed into chronological context, which will be addressed with developments in geochronology and paleoecology together with increasingly powerful computational tools to estimate the age of samples using a molecular clock. The resulting deep-time DNA will enable increasingly detailed reconstruction of evolutionary history across repeated environmental perturbations, refining understanding of adaptive evolution, community organization, and ecosystem resilience. Moreover, as the past by its nature is different from anything that exists today, access to deep-time DNA provides ample yet unpredictable opportunities for scientific discovery.

## Box 1: Deep-time mammoth DNA and the inference of a lineage’s entire evolutionary story

The power of a deep-time genomic approach was showcased in a study by van der Valk and colleagues (10) in which genome-wide data collected from three Siberian mammoths, dated to approximately 700 ka to 1.2 Ma, made it possible to examine mammoth evolution from multiple genomic angles:

### A new lineage:

The oldest of the mammoth specimens, Krestovka, belonged to a previously unknown and divergent evolutionary lineage. This implied that two distinct lineages of mammoth, Krestovka and the ancestors of woolly mammoths, lived in Siberia during the later stages of the Early Pleistocene. The analysis also revealed that mammoths belonging to the Krestovka lineage were the first mammoths to colonize North America 1.5–1.2 Ma (57).

### Hybridization:

Multiple lines of evidence suggested that the Columbian mammoth originated as a result of hybridization between the Krestovka lineage and early woolly mammoths. This hybridization took place as woolly mammoths expanded into North America during the Middle Pleistocene, after the Krestovka lineage was already established on the continent (Fig. 3). Columbian mammoths derive approximately 50% ancestry from each of these two lineages.

### Adaptive evolution:

The deep-time nature of the mammoth data set allowed van der Valk et al to estimate the rate of adaptive evolution in mammoths. Their results suggested that the evolutionary origin of the woolly mammoth lineage did not coincide with an increased rate of protein-coding changes and therefore higher rates of positive selection across the genome (10). Subsequent analyses identified a suite of genes that underwent protein-coding changes during the last 700 ka and were thus unique to woolly mammoths (37).
